# Beyond PlayerLoad: Detection of Critical Moments and Injury Risk in Elite Women’s Futsal

**DOI:** 10.3390/sports14010008

**Published:** 2026-01-01

**Authors:** Diego Hernán Villarejo-García, Carlos Navarro-Martínez, José Pino-Ortega

**Affiliations:** 1 BioVetMed & SportSci Research Group, Faculty of Sport Sciences, University of Murcia, 30720 San Javier, Spain; dvillarejo@um.es (D.H.V.-G.); josepinoortega@um.es (J.P.-O.); 2 Education, Diversity and Quality Research Group, Faculty of Education, University of Murcia, Campus de Espinardo, 30100 Murcia, Spain

**Keywords:** accelerations and decelerations, contextual variables, elite women’s futsal, external load monitoring, injury prevention

## Abstract

Monitoring the volume and intensity of physical load is essential in elite women’s futsal to optimize performance and prevent injuries. However, external load indicators such as PlayerLoad may underestimate critical moments in competition where the intensity and volume of accelerations and decelerations sharply increase. This study aimed to identify and characterize such critical moments by analyzing the interaction between current score, playing position, match half, and location on acceleration and deceleration volume (distance, km/h) and intensity (peak, m/s^2^). Thirteen elite female futsal players (age: 29.9 ± 5.1 years; height: 164.96 ± 4.22 cm; body mass: 60.31 ± 4.56 kg) competing in the Spanish First Division were analyzed over a full season. All match accelerations and decelerations recorded with WIMU PRO™ inertial devices were processed using four Linear Mixed Models (LMMs). Significant interactions emerged across all models. Volume increased when winning, particularly among pivots, while intensity rose during adverse conditions, especially when losing at home. Interindividual variability was minimal (ICC < 1%). Physical load in women’s futsal follows two situational patterns: volume increases when leading, and intensity peaks when trailing. Identifying these critical moments provides insight beyond total load metrics, offering guidance for individualized and context-specific injury prevention.

## 1. Introduction

Game actions involving high-intensity accelerations and sudden decelerations have been reported as injury risk factors in team sports [1]. This may be due to the high mechanical load (magnitude and frequency) to which the neuromuscular system is exposed during such actions [2]. While accelerations require maximal concentric force production, decelerations pose a greater injury risk [3]. These actions involve high-intensity eccentric contractions necessary to absorb body inertia during braking. In team sports, this has been identified as a key injury mechanism, particularly affecting the hamstring muscles [4]. In addition to high intensity, another major risk factor is the large accumulated load volume that these types of actions entail during competition [5]. The combination of high intensity and large volume may create vulnerability scenarios for both acute and overuse injuries in athletes [6], making it essential to monitor and control the external load to which they are exposed.

To monitor and control external load in team sports, metrics such as PlayerLoad (PL) have been used [7]. PL is calculated from triaxial accelerometer data that detect acceleration in three spatial planes [8]. Through a mathematical algorithm, the sum of the rates of change in acceleration and deceleration at each instant is computed [9]. In this way, a general index of the total mechanical load experienced by an athlete is obtained [10]. This metric has important practical value for coaches, athletes, and strength and conditioning staff, as it expresses in a single value the total external load from all accelerations and decelerations performed. However, PL also presents certain limitations [11]. One of these is that PL averages load over time, which prevents the detection of peaks in intensity and volume at specific moments. Identifying these peaks is crucial to understanding the situational factors under which they occur [12,13].

Although still limited compared with men’s futsal, some studies have described external load using the PL index in women’s futsal. The application of PL has made it possible to establish load profiles. For example, a study with 12 professional Spanish players reported a relative PL of 10.5 Arbitrary Units (AU)·min^−1^ and observed a significant decrease in this load and in high-intensity accelerations (>2 m/s^2^) during the second half of matches [9]. Another investigation with 10 elite Portuguese players found a high volume of accelerations and decelerations > 2 m/s^2^ and showed that this load increased when competing against higher-level opponents [7]. Likewise, another study [10] measured PL across different joints during linear treadmill running in elite futsal players, showing large differences in load between body segments and between individuals. Although these studies characterize external load in women’s futsal, they focus primarily on describing general physical demands or isolated contextual factors influencing PL. This approach is valuable for planning and monitoring workload; however, it does not capture how multiple situational variables interact simultaneously to create specific load peaks and moments of heightened injury risk.

To address this gap, descriptive, retrospective, and analytical research designs can be implemented [14] to analyze physical load data in competition using statistical approaches capable of handling complex interactions. In this regard, the Linear Mixed Model (LMM) is one of the most suitable tools for this purpose [15]. Unlike univariate or bivariate analyses, LMMs can model the effects of multiple factors (e.g., match location, score, and playing position) on external load indices—such as the volume and intensity of accelerations and decelerations—while accounting for the nested data structure involving multiple actions per player. This statistical approach also quantifies individual variability [16], determining the proportion of performance variance explained by stable differences between players versus situational factors. This enables understanding whether load profiles represent a personal trait or a universal response to game context.

From a tactical perspective, match status acts as a constraint on physical performance [7]. It has been suggested that when trailing, teams tend to increase their offensive and defensive intensity to reverse the score, adopting strategies such as high pressing or faster transitions [9]. This tactical need to ‘chase the game’ theoretically imposes a higher physical load compared to match statuses where the team is winning or drawing and might adopt a more conservative possession-based style [10].

Therefore, the purpose of this study was to determine the influence of the interaction between current score, playing position, match location, and match half on the volume (distance) and intensity (peak value) of accelerations and decelerations in elite women’s futsal players. The authors hypothesized that the volume of actions would be greater in losing situations, whereas the intensity of actions would be higher during moments of tactical adversity—such as losing, playing at home, or during the second half of the match. This type of research aims to identify dynamic, context-dependent risk profiles and critical moments of competition that are of utmost importance for athlete health and injury prevention. Such knowledge will enable coaches and conditioning professionals to design more precise monitoring and training strategies, tailored not only to individual players but also to the specific demands of each competitive context.

## 2. Materials and Methods

### 2.1. Participants

The study sample consisted of 13 elite female futsal players (3 pivots, 2 wing–pivots, 3 defenders, 2 wing–defenders, and 3 wings) (age: 29.9 ± 5.1 years; height: 1.65 ± 0.04 m; body mass: 60.31 ± 4.56 kg) who competed in the Primera División of the Royal Spanish Football Federation during the 2020/2021 season. Data were collected from 23 official league matches. Given the elite professional setting, the sample size was determined by the total number of players in the squad (census sampling), relying on the high volume of repeated longitudinal observations to ensure statistical power.

All participants were professional athletes with at least five years of experience in elite futsal and had no musculoskeletal injuries or physical limitations that could have affected their performance during the study period.

In accordance with the Declaration of Helsinki [17] and to ensure player confidentiality, all performance data were anonymized prior to analysis. The study protocol was approved by the Ethics Committee of the University of Murcia (approval number: 3180/2020). All players were informed about the experimental protocol and its potential benefits during a meeting held before the start of the study and provided written informed consent to voluntarily participate.

### 2.2. Procedure

**Figure 1:**
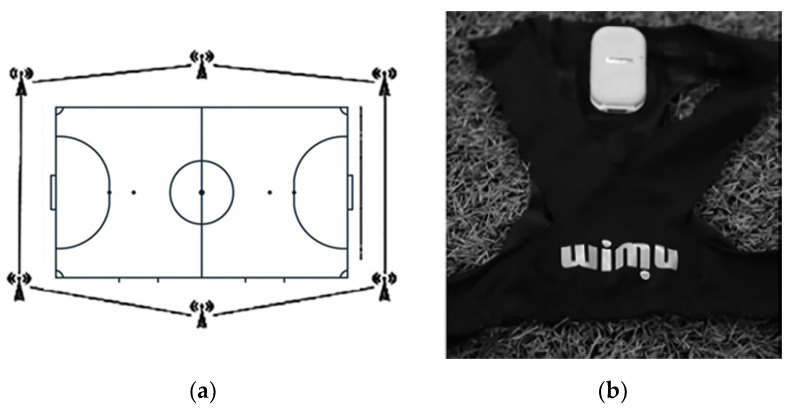
Equipment setup for data collection: (**a**) placement of the antennas around the futsal court; (**b**) specific anatomical harness used to secure the inertial device.

The study design was observational, descriptive, and cross-sectional [14], adhering to the STROBE guidelines (Supplementary Material). To analyze the variables peak acceleration, peak deceleration, distance acceleration, and distance deceleration, WIMU PRO^TM^ inertial devices (RealTrack Systems, Almería, Spain) were used. These devices were equipped with sensors and antennas that allowed indoor data collection through an Ultra-Wide Band (UWB) chip. A system of six UWB antennas was placed around the perimeter of the futsal court (Figure 1a).

The data quality of the device showed an average difference of 0.86% and 0.44% for the “x” and “y” coordinates, respectively, when compared to the official field dimensions (40 m × 20 m). The inter-device reliability demonstrated intraclass correlation coefficient (ICC) values of 0.82 for the “x” coordinate and 0.73 for the “y” coordinate (two-way mixed effects, absolute agreement). Synchronization was carried out using ANT+ technology.

The inertial devices were positioned between the players’ scapular areas within a specific anatomical harness (Figure 1b). Each player was equipped 30 min before the start of the match. Only data corresponding to active playing periods during official competition were included in the analysis.

Data processing was performed using the SPRO^TM^ software, version 989 (RealTrack Systems, Almería, Spain). To ensure that the analysis focused exclusively on competitive demands, all data corresponding to warm-up periods, breaks (halftime or time-outs), and intervals when players were on the bench for technical decisions were excluded from the dataset.

### 2.3. Variables

In the present research, the following independent and dependent variables were analyzed:

#### 2.3.1. Dependent Variables

- Peak Acceleration: Defined as the maximum rate of change in velocity reached during a single action, measured in meters per second squared (m/s^2^). This variable represents the intensity of each physical effort. Peak Deceleration: Defined as the minimum rate of change in velocity reached during a single action, measured in meters per second squared (m/s^2^). This variable also represents the intensity of each physical effort.
- Distance Acceleration: Defined as the distance (in meters) covered during a single acceleration action. This variable represents the volume of each physical effort. Distance Deceleration: Defined as the distance (in meters) covered during a single deceleration action. This variable also represents the volume of each physical effort.

#### 2.3.2. Independent Variables

Four categorical independent variables were included to contextualize performance:

- Current Score: Defined as the match score status at the moment each action occurred. It was classified into three levels: Winning, Losing, and Drawing.
- Position: Defined as each player’s main tactical role within the team. Positions were classified into five roles: Defense, Pivot, Wing, Wing–Defense, and Wing–Pivot.
- Halves: Defined as the period of the match in which the action occurred. It was divided into two levels: First and Second.
- Location: Defined as the venue where the match was played. It was classified into two levels: Home and Away.

### 2.4. Statistical Analysis

The four dependent variables (peak acceleration, peak deceleration, distance acceleration, and distance deceleration) showed a skewed distribution and were log10-transformed prior to analysis to meet normality assumptions. A Linear Mixed Model (LMM) was applied to each dependent variable due to the hierarchical nature of the data, where repeated measurements were nested within each player.

The model included the independent variables Current Score, Position, Halves, and Location as fixed effects, along with all two-, three-, and four-way interactions. A random intercept for players was specified to model individual variability among participants. The inclusion of a random intercept for players allowed the model to account for stable inter-individual characteristics (e.g., age, experience, or physical fitness) without reducing statistical power by adding them as fixed effects.

Subsequently, a backward elimination procedure was applied to obtain a more parsimonious model. Starting from the full model, non-significant higher-order interaction terms were sequentially removed. The decision to retain or eliminate a term was based on the Likelihood Ratio Test (LRT), comparing models with and without the given term. A term was removed if its exclusion did not result in a significant reduction in model fit (*p* > 0.05). In accordance with the principle of hierarchy, all main effects and lower-order interactions involved in a significant retained interaction were kept in the final model, regardless of their individual *p*-value.

Statistical assumptions were verified. Linearity and homoscedasticity of residuals were visually inspected and deemed acceptable. Visual examination of residual plots and Q–Q plots confirmed adequate linearity and homoscedasticity. Although formal normality tests were significant due to the large sample size, the distribution of transformed residuals was considered practically acceptable.

A significance level of *p* < 0.05 was established for fixed effects and interactions. To determine the practical significance of the effects, Partial Eta Squared (η^2^*p*) was calculated, with values interpreted as small (0.01), medium (0.06), and large (0.14) [18]. For detailed interpretation of significant interactions, Estimated Marginal Means (EMMs) were analyzed.

All statistical analyses were performed using Jamovi, version 2.6 [19], the GAMLj module (version 3.6.5), and R Studio, version 4.5.0 [20].

### 2.5. Data Availability

The data used for statistical analysis and the results of this work are publicly available in the Research Data Repository (DIGITUM) under the following Digital Object Identifier (DOI): http://hdl.handle.net/10201/163729 (accessed on 26 September 2025).

## 3. Results

The following section presents the descriptive results of the performance variables and the main findings from the four adjusted Linear Mixed Models (LMMs). Specifically, Table 1 displays the descriptive statistics (number of actions, mean, standard deviation, and range) for the four dependent variables analyzed. Data are shown for the total sample and are detailed according to each playing position.

As shown in Table 1, a total of 17,938 accelerations and 18,815 decelerations were analyzed. This descriptive analysis indicates that the Wing–Defender position recorded the highest Peak Acceleration values (M = 9.20 m/s^2^), whereas the Pivot position recorded the lowest Peak Acceleration values (M = 8.36 m/s^2^). This pattern was also observed for Peak Deceleration, where the Pivot position showed the lowest mean value (M = 8.29 m/s^2^).

Regarding the volume of actions, Wing–Defenders covered the greatest Distance Acceleration (M = 4.13 m). Conversely, Pivots covered the shortest Distance Acceleration (M = 3.32 m) and Distance Deceleration (M = 3.21 m).

Table 2 presents a summary of the fixed effects and statistically significant interactions from the Linear Mixed Models that analyzed the intensity variables (Peak Acceleration and Peak Deceleration).

As detailed in Table 2, regarding the main effects, being in a winning situation was associated with an increase in Peak Acceleration (β = 0.033) and Peak Deceleration (β = 0.040) compared to a drawing situation. In addition, the second half of the match was associated with a significant reduction in Peak Acceleration (β = −0.012).

An interaction between Current Score and Location was also observed, where losing at home significantly increased Peak Acceleration (β = 0.066) and Peak Deceleration (β = 0.077). Furthermore, a specific interaction between Halves and Position was identified, in which Pivots showed a reduction in Peak Deceleration during the second half (β = −0.056).

Finally, the strongest effect was a three-way interaction: losing at home produced the most pronounced response in Pivots, both for Peak Acceleration (β = 0.100) and Peak Deceleration (β = 0.129).

Table 3 summarizes the fixed effects and statistically significant interactions from the Linear Mixed Models that analyzed the volume variables (Distance Acceleration and Distance Deceleration).

As shown in Table 3, being in a winning situation compared to drawing was associated with an increase in both Distance Acceleration (β = 0.122) and Distance Deceleration (β = 0.103). Regarding Position, being a Pivot was associated with shorter distances compared to Defenders.

A strong interaction between Current Score and Position was also observed. The increase in distance when winning was significantly more pronounced in Pivots, for both Distance Acceleration (β = 0.091) and Distance Deceleration (β = 0.097). This pattern was also evident in Wing–Defenders, who showed significant increases in Distance Acceleration (β = 0.071) and Distance Deceleration (β = 0.084) when winning.

**Figure 2:**
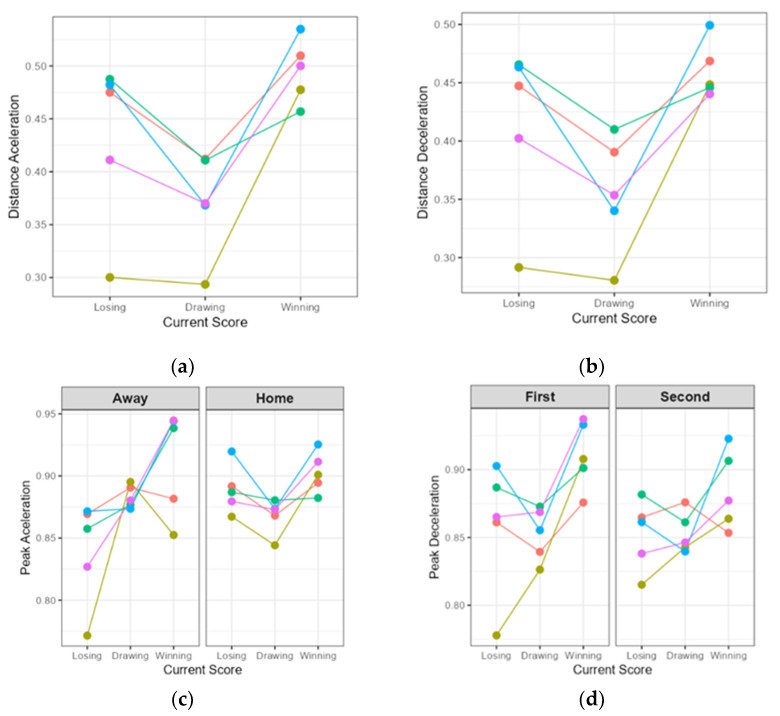
Interaction effects between contextual variables and effort volume and intensity. Interaction effects between partial score, playing position, and contextual factors (location and match half) on effort volume and intensity. (**a**) Distance Acceleration; (**b**) Distance Deceleration; (**c**) Peak Acceleration, separated by match location; (**d**) Peak Deceleration, separated by match half. Dots represent estimated marginal means, and lines connect values for each position to facilitate visualization of the interaction. Line colors represent playing positions: cyan for Defenders, red for Pivots, olive for Wings, green for Wing–defenders, and purple for Wing–Pivots.

Figure 2 presents the estimated marginal means for the independent variables across the different playing positions.

As shown in Figure 2, the analysis of Distance Acceleration (Figure 2a) and Distance Deceleration (Figure 2b) revealed a significant interaction between Current Score and Position. This effect was significant for both Distance Acceleration (F(8, 9482.44) = 13.70, *p* < 0.001, η^2^*p* = 0.011) and Distance Deceleration (F(8, 9010.84) = 12.50, *p* < 0.001, η^2^*p* = 0.011). As can also be seen in Figure 2a, this increase was significantly greater for Pivots than for Defenders (β = 0.091, *p* < 0.001) when winning. This trend is similarly reflected in Figure 2b, where Pivots showed higher Distance Acceleration compared to Defenders (β = 0.096, *p* < 0.001) when winning.

In contrast, the analysis of Peak Acceleration (Figure 2c) and Peak Deceleration (Figure 2d) revealed patterns modulated by match context (Location and Halves). For Peak Acceleration, a significant three-way interaction was found between Current Score, Position, and Location (F(8, 5257.39) = 2.67, *p* = 0.006, η^2^*p* = 0.004). This response to Current Score was led by the Pivot position when losing (β = 0.099, *p* = 0.001).

The results of the fourth statistical model (Figure 2d) also showed a tendency to model Peak Deceleration responses according to Location (F(8, 18,755) = 3.27, *p* < 0.001, η^2^*p* = 0.001) and Halves (F(4, 18,755) = 4.21, *p* = 0.002, η^2^*p* = 0.001). Specifically, Wing–Defenders (β = −0.051, *p* = 0.007) and Wing–Pivots (β = −0.056, *p* < 0.001) significantly reduced Peak Deceleration intensity during the Second Half compared with Defenders. However, Pivots showed a tendency to increase Peak Deceleration when losing in the Second Half (β = 0.071, *p* = 0.026).

The analysis of random components across the four models revealed that the variability attributable to individual differences among players was minimal. For distance variables, the Intraclass Correlation Coefficient (ICC) accounted for 0.47% of the total variance for acceleration and 0.40% for deceleration. For Peak Acceleration and Peak Deceleration variables, ICC values were 0.01% for acceleration.

## 4. Discussion

The aim of this study was to determine the influence of situational variables (score, position, location, and match half) on the volume and intensity of accelerations and decelerations in elite women’s futsal. The findings reveal two distinct and opposing contextual load profiles: contrary to our initial expectations, effort volume significantly increased during advantageous situations (winning), whereas intensity metrics peaked during moments of tactical adversity. These results suggest a complex competitive environment where load accumulation and acute intensity spikes are driven by opposing match statuses, with minimal interindividual variability (ICC < 1%) attributable to players.

The findings partially confirmed these hypotheses, revealing two distinct and well-defined contextual load profiles. Contrary to expectations, effort volume increased during winning situations, whereas intensity, as hypothesized, increased during moments of tactical adversity. Regarding interindividual variability, the results showed that only a minimal portion of the variance in these actions was attributable to differences between players (ICC < 1%). Overall, these findings describe a situational context in elite women’s futsal that cannot be explained by general load metrics such as PlayerLoad (PL). This type of approach may represent a valid methodology for detecting periods of elevated injury risk during elite women’s futsal matches [21].

A deeper analysis of the main results, particularly regarding effort volume (Figure 2a,b), refuted the first hypothesis proposed by the authors. Specifically, the results revealed that the distance of accelerations and decelerations significantly increased when winning (Current Score × Position interaction, *p* < 0.001, η^2^*p* = 0.011). This counterintuitive finding suggests that load volume may not be driven by adverse match situations, but rather by favorable ones. One possible explanation is that when leading on the scoreboard, the opposing team takes greater tactical risks, creating larger spaces that allow for longer runs and counterattacks. Another plausible explanation is that, when leading, teams may spend longer defending against the use of a “flying goalkeeper,” increasing cumulative defensive load.

This pattern was primarily led by Pivots, who increased their acceleration distance by 23.3% (β = 0.091, *p* < 0.001) and their deceleration distance by 24.7% (β = 0.096, *p* < 0.001) under this condition. From an injury risk perspective, this implies that periods of apparent control in the match are, in fact, moments of high accumulated load for players. This discovery could be used by coaches to modulate the workload of players exposed to such critical periods during the match or throughout the training week.

Furthermore, sports psychology literature offers a complementary explanation for these findings through the Psychological Momentum (PM) theory. PM is defined as a force that changes behavior and performance outcomes based on perceived progress toward a goal [22]. Research suggests that positive momentum (winning) enhances collective efficacy and task cohesion, encouraging players to maintain higher levels of exerted effort (volume) to proactively secure the victory. In contrast, negative momentum (losing) has been associated with cognitive anxiety and performance deterioration, often triggering disorganized, high-intensity ‘emergency’ efforts (peaks) rather than the sustained volume associated with controlling the game flow [23].

In contrast, and consistent with the second hypothesis, the analysis of intensity (Figure 2c,d) identified adversity as the main modulating factor. The most relevant finding was a three-way interaction that identified losing at home as the “Critical Moment” of highest intensity (*p* = 0.006, η^2^*p* = 0.004). This response was again led by Pivots, who increased their Peak Acceleration by 25.9% (β = 0.100, *p* < 0.05) and their Peak Deceleration by 34.6% (β = 0.129, *p* < 0.05) under this condition.

Additionally, the analysis of deceleration intensity revealed clear fatigue management through the Position × Halves interaction (*p* = 0.002, η^2^*p* = 0.001), where Wing players reduced their intensity during the second half by up to 12.1% (e.g., Wing–Pivot: β = −0.056, *p* < 0.001). In this scenario, a fatigued player performing maximal-intensity actions represents an acute muscle injury risk situation due to excessive load [24]. It is possible that this trend of increasing acceleration and deceleration volume and intensity is even more pronounced during the second halves and in the final minutes of matches when teams are defending against a flying goalkeeper—an aspect not considered in this study.

An important finding of this study, made possible through the use of LMMs, was the minimal variability in performance attributable to individual differences among players. In elite sport—where differences in physical, technical, and tactical capacity are expected—we anticipated a considerable degree of interindividual variability. However, our results indicate that this is not the case for these high-intensity actions. This may suggest that the identified Critical Moments impose a situational demand that homogenizes the physical response. Specifically, the volume and intensity requirements of accelerative and decelerative actions associated with match context appear to minimize individual characteristics (such as fitness level or technical ability), forcing all players into a similar physical effort pattern to cope with these game situations.

The main implication of this finding is that risk during these critical moments is not an inherent characteristic of certain “fragile” players but rather a direct consequence of the game situation itself. Therefore, any elite player exposed to combinations of score status, fatigue, and location would be subject to a similar acute risk profile, emphasizing the importance of managing game situations as carefully as player workload.

Due to the limited number of scientific studies in women’s futsal examining PlayerLoad, accelerations, and decelerations, comparisons are challenging. Nevertheless, some research has quantified external load volume in women’s futsal, reporting relative distances and PlayerLoad values similar to those described previously [7]. However, to our knowledge, no studies have examined complex contextual interactions influencing load volume during competition. Consequently, the results of this study are both novel and counterintuitive, showing that volume increases when winning rather than when losing. Specifically, our results demonstrate that being ahead on the scoreboard significantly increases the distance of accelerations and decelerations compared to a drawing situation. This finding contrasts with previous studies that have associated high-demand scenarios—such as competing against higher-level opponents—with a general increase in load [7].

Regarding intensity, our results align with the notion that moments of adversity amplify physical demands. The finding that losing at home represents the highest-intensity scenario for both acceleration and deceleration complements previous studies in elite men’s futsal, which identified 30 s periods of play with a high number of intense accelerations and decelerations per minute [25]. However, those studies did not identify the contextual factors driving these intensity peaks. In our study, this condition was led by Pivots, who showed substantial increases in both Peak Acceleration and Peak Deceleration. Therefore, this study provides specific evidence demonstrating that behind a single total PlayerLoad value lie two opposing, context-dependent load patterns—an insight that is crucial for effective injury prevention strategies.

While absolute external load values are generally higher in male sports due to biological differences in power and speed, situational dynamics appear to share similarities. For instance, research in elite male futsal has also identified that high-intensity acceleration efforts often cluster with adverse results, possibly due to the need to regain possession to score [26]. This has also been observed in sports such as handball, where ‘critical moments’ characterized by close scorelines have been linked to spikes in neuromuscular variability and intensity, regardless of gender [27]. However, a distinctive finding of our study is the clear dissociation between volume and intensity. Unlike previous literature [26,27], where both variables tend to fluctuate simultaneously, our female cohort exhibited a distinct tactical strategy. Specifically, increased volume was observed when winning, and increased intensity when losing. This highlights the evidence of different tactical, technical, and physical strategies depending on the specific context.

The practical implications of these findings may help improve several aspects of training and injury prevention. On one hand, it can be suggested that load monitoring should include the contextual aspects of competition. This means not only analyzing total match values in metrics such as PlayerLoad, since these may mask risk peaks that occur at specific moments. For this to be practically applicable, real-time and on-site monitoring of accelerations and decelerations is necessary, requiring the development of software capable of automatically detecting these Critical Moments and sending actionable alerts to coaches, strength and conditioning staff, or athletes.

Moreover, training and physical preparation programs should consider the specificity of acceleration and deceleration volume and intensity according to playing position and the identified high-risk situations. Knowing that Pivots are exposed to higher-intensity loads than other positions when losing at home or during the second half, coaches can design drills that replicate these demands. Specific conditioning programs could also be implemented for these players to better withstand high acute loads under fatigue conditions.

Finally, there is a clear need for real-time awareness of intensity and volume peaks. For instance, technology developers could integrate alert systems for players exposed to prolonged Critical Moments. These alerts could support strategic substitutions aimed at reducing injury likelihood. Ultimately, it is necessary to develop a comprehensive training and competition system in which IMU-based technology provides data, software interprets it, and coaches and physical trainers make informed decisions regarding volume and intensity increases during competition. This approach transforms prevention from reactive to proactive, using contextual analysis to protect athlete health during periods of greatest vulnerability.

This study presents several limitations that should be considered. The analysis was conducted on a single team, which limits the generalizability of the results. Due to the sample size limitations, the present findings should be considered as a preliminary analysis of contextual load in women’s futsal. Future investigations with expanded samples and multi-team designs are required to replicate and validate these observed patterns. Additionally, although key situational variables were controlled, the observational design did not differentiate between specific tactical phases of play, such as “flying goalkeeper” situations, opponent team level, or different offensive (e.g., 4–0 vs. 3–1) and defensive systems (e.g., high press vs. low block). These factors could act as moderators of the effects observed.

Finally, although the effect sizes were statistically significant, they were modest, and no internal load variables were included. Likewise, individual physiological factors such as menstrual cycle phase [28] and psychological factors such as competitive stress [29] were not considered, even though these may modulate physical responses and injury vulnerability.

Future research should replicate this analysis in other populations and include these additional variables to determine whether Critical Moments become more pronounced or attenuated depending on the game system employed or the quality of the opponent. Including internal load variables would also provide a more comprehensive understanding of the physiological stress experienced by players.

Lastly, although the logarithmic transformation substantially improved the distribution of residuals, formal normality tests remained significant. This finding is attributed to the large sample size, which increases the sensitivity of statistical tests. Since visual inspection of the Q–Q plots indicated practical acceptability and LMMs are robust to minor deviations from normality, the validity of the findings was not considered compromised.

## 5. Conclusions

Physical load in elite women’s futsal follows two opposing patterns. While the volume of accelerations and decelerations increases during advantageous situations, intensity peaks during disadvantageous moments. By analyzing this opposing relationship, the present study proposes a methodology that goes beyond PlayerLoad (PL)—specifically, by using acceleration and deceleration indices to objectively identify and quantify these Critical Moments of the game. This new perspective is essential for developing advanced injury prevention strategies, enabling sports professionals to move from general load monitoring to context-specific risk management during the most demanding phases of competition.

## Figures and Tables

**Table 1:** Descriptive statistics for intensity and volume variables.

	Peak Acceleration (m/s^2^)					Peak Deceleration (m)				
Position	N	M	SD	95% CI		N	M	SD±	95% CI	
				Min	Max				Min	Max
General	17,938	8.76	4.55	1.29	35.9	18,815	8.68	4.64	1.00	3.66
Defender	3725	8.91	4.55	1.37	29.8	4008	8.61	4.53	01.01	2.56
Pivot	5049	8.36	4.60	1.29	34.1	5099	8.29	4.75	1.00	3.66
Wing	2384	8.83	4.43	1.45	26.9	2478	8.99	4.50	1.19	2.87
Wing–defender	2453	9.20	4.47	1.53	24.2	2629	09.07	4.53	1.13	2.98
Wing–Pivot	4327	8.81	4.65	1.32	35.9	4601	8.79	4.67	1.00	3.66
	**Distance Acceleration**					**Distance Deceleration**				
Position	N	M	SD	95% CI		N	M	SD±	95% CI	
				Lower	Upper				Lower	Upper
General	17,938	3.78	03.9	0.07	24.6	18,815	3.55	2.86	0.02	2.14
Defender	3725	04.1	3.14	0.07	24.6	4008	3.72	2.87	0.11	2.08
Pivot	5049	3.32	2.93	0.12	21.4	5099	3.21	2.84	0.06	1.88
Wing	2384	3.91	03.3	0.11	19.6	2478	3.72	2.76	0.11	1.83
Wing–defender	2453	4.13	3.25	0.17	23.7	2629	3.83	2.94	0.03	1.87
Wing–Pivot	4327	3.83	3.20	0.07	22.3	4601	3.54	2.87	0.02	2.14

Note: N: Number; M: Mean; SD: Standard Deviation; 95% CI: Confidence Interval; Min: Minimum; Max: Maximum.

**Table 2:** Summary of fixed effects and interactions for intensity variables.

	Peak Acceleration			Peak Deceleration		
Predictor	(β)	95% CI		(β)	95% CI	
		Min	Max		Min	Max
CS: Winning vs. Drawing	0.033 *	0.022	0.044	0.040 *	0.024	0.055
H: Second Halve vs. First Halve	−0.012 *	−0.023	−0.001	−0.010 *	−0.021	0.001
CS: Losing vs. L: Home	0.066 *	0.042	0.089	0.077 *	0.053	0.101
P: Pivot vs. H: Second Half	−0.030 *	−0.061	0.001	−0.056 *	−0.087	−0.025
CS: Losing vs. P: Pivot vs. L: Home	0.100 *	0.039	0.161	0.129 *	0.066	0.191

Note: β: estimated coefficient; 95% CI: 95% confidence interval; *: *p* < 0.05; CS: Current Score; H: Halves; L: Location; P: Position; Min: Minimum; Max: Maximum.

**Table 3:** Summary of fixed effects and interactions for volume variables.

	Distance Aceleration			Distance Deceleration		
Predictor	(β)	95% CI		(β)	95% CI	
		Min	Max		Min	Max
CS: Winning vs. Drawing	0.122 *	0.106	0.139	0.103 *	0.087	0.119
P: Pivot vs. Defense	−0.097 *	−0.155	−0.039	−0.080 *	−0.134	−0.026
CS: Winning vs. P: Pivot	0.091 *	0.046	0.137	0.097 *	0.053	0.140
CS: Winning vs. P: Defense_Pivot	0.071 *	0.013	0.128	0.084 *	0.029	0.140

Note: β: estimated coefficient; 95% CI: 95% confidence interval; *: *p* < 0.05; CS: Current Score; P: Position; Min: Minimum; Max: Maximum.

## Data Availability

The data supporting the findings of this study are openly available in the Research Data Repository (DIGITUM) at the University of Murcia under the DOI: http://hdl.handle.net/10201/163729 (accessed on 26 September 2025).

## Supplementary Materials

The following supporting information can be downloaded at: https://www.mdpi.com/article/10.3390/sports14010008/s1.

## Abbreviations

The following abbreviations are used in this manuscript:

AU	Arbitrary Units
COD	Change of Direction
EMMs	Estimated Marginal Means
GRF	Ground Reaction Force
HR	Heart Rate
ICC	Intraclass Correlation Coefficient
IMU	Inertial Measurement Unit
LMM	Linear Mixed Model
LRT	Likelihood Ratio Test
PL	PlayerLoad
