# Supplementary material for: Beyond PlayerLoad: Detection of Critical Moments and Injury Risk in Elite Women’s Futsal
Source: Sports (Basel). 2026 Jan 1;14(1):8. doi: 10.3390/sports14010008 (PMC12845870; doi:10.3390/sports14010008)
Supplement: Supplementary file 1 [file sports-14-00008-s001.zip › sports-4001129-supplementary.pdf]

STROBE Statement—Checklist of items that should be included in reports of ***cross-sectional studies***

	Item No	Recommendation
**Title and abstract**	1	(*a*) Title Page (Observational design is implied; explicitly stated in Abstract)
		(*b*) Abstract, lines 19-28
**Introduction**		
Background/rationale	2	Section 1. Introduction, paragraphs 1-6
Objectives	3	Section 1. Introduction, last paragraph
**Methods**		
Study design	4	Section 2.2 Procedure (moved from 2.4), paragraph 1 ("observational, descriptive, and cross-sectional")
Setting	5	Section 2.1 Participants, paragraph 1 (League details, season 2020/2021)
Participants	6	(*a*) Section 2.1 Participants, paragraph 1 (Inclusion criteria, experience level)
Variables	7	Section 2.3 Variables (moved from 2.2)
Data sources/ measurement	8*	Section 2.2 Procedure, paragraphs 1-3 (WIMU PRO specifications and setup)
Bias	9	Section 2.2 Procedure, last paragraph (Exclusion of warm-ups/bench time to avoid selection bias)
Study size	10	Section 2.1 Participants, paragraph 1. Text added: "Given the elite professional setting, the sample size was determined by the total number of players in the squad (census sampling), relying on the high volume of repeated longitudinal observations to ensure statistical power."
Quantitative variables	11	Section 2.4 Statistical Analysis, paragraph 1 (Log-transformation)
Statistical methods	12	(*a*) Section 2.3 Statistical Analysis, paragraph 2. (Use of Linear Mixed Models with fixed effects for contextual variables and random effects for players to control for individual variability).
		(*b*) Section 2.3 Statistical Analysis, paragraph 2 (Inclusion of two-, three-, and four-way interactions) and paragraph 4 (Use of Estimated Marginal Means - EMMs for interaction analysis).
		(*c*) Section 2.2 Procedure, last paragraph. (Data cleaning process: exclusion of warm-ups, breaks, and bench time to ensure only active data was analyzed; LMMs handle unbalanced data naturally)
		(*d*) Section 2.3 Statistical Analysis, paragraph 2. (Hierarchical structure modeled via LMM: repeated measures nested within players).
		(*e*) Section 2.3 Statistical Analysis, paragraph 2. (Backward elimination procedure and Likelihood Ratio Test - LRT to compare model fit and select the most parsimonious model).
**Results**		
Participants	13*	(a) Section 2.1 Participants (N=13 players) and Section 3 Results, paragraph 2 (Total actions analyzed: 17,938 accelerations and 18,815 decelerations).
		(b) Section 2.2 Procedure, last paragraph (Data exclusion criteria: warm-up periods, breaks, and bench time were excluded to analyze only active play).
		(c) Not applicable (Single cohort, no drop-outs reported for the analysis period).
Descriptive data	14*	(a) Section 2.1 Participants (Demographics: Age, Height, Body Mass) and Table 1 (Descriptive statistics by playing position).
		(b) Section 3 Results, Table 1 (Reports 'N' of actions for each position; Linear Mixed Models handled unbalanced data structure).

Outcome data	15*	Table 1 (Means, Standard Deviations, and Confidence Intervals for Peak/Distance Acceleration and Deceleration) .
Main results	16	(*a*) Tables 2 and 3 (Adjusted estimates from Linear Mixed Models: β coefficients, 95% Confidence Intervals, and p-values for fixed effects and interactions).
		(*b*) Not applicable (Continuous outcome variables were Log-transformed, not categorized)
		(*c*) Not applicable.
Other analyses	17	Section 3 Results, paragraphs 3-6 (Interaction analyses visualized in Figure 2) and last paragraph (ICC analysis for inter-individual variability).
**Discussion**		
Key results	18	Section 4 Discussion, paragraph 1 (Summary of findings vs. hypotheses)
Limitations	19	Section 4 Discussion, paragraphs 13-14 (Single team, lack of tactical phase differentiation, lack of internal load, small effect sizes)
Interpretation	20	Section 4 Discussion, paragraphs 2-8 (Interpretation of volume increasing when winning and intensity increasing when losing; practical implications)
Generalisability	21	Section 4 Discussion, paragraph 13 (Acknowledges limitations of generalizing from a single elite team)
**Other information**		
Funding	22	Funding Section (Statement: "This research received no external funding").

*Give information separately for exposed and unexposed groups.

Note: An Explanation and Elaboration article discusses each checklist item and gives methodological background and published examples of transparent reporting. The STROBE checklist is best used in conjunction with this article (freely available on the Web sites of PLoS Medicine at http://www.plosmedicine.org/, Annals of Internal Medicine at http://www.annals.org/, and Epidemiology at http://www.epidem.com/). Information on the STROBE Initiative is available at www.strobe-statement.org.
